# Cross-Sectional Study of Resilience, Positivity and Coping Strategies as Predictors of Engagement-Burnout in Undergraduate Students: Implications for Prevention and Treatment in Mental Well-Being

**DOI:** 10.3389/fpsyt.2021.596453

**Published:** 2021-02-16

**Authors:** Jesús de la Fuente, Flavia H. Santos, Angélica Garzón-Umerenkova, Salvatore Fadda, Giuliana Solinas, Silvia Pignata

**Affiliations:** ^1^School of Education and Psychology, University of Navarra, Pamplona, Spain; ^2^School of Psychology, University of Almería, Almería, Spain; ^3^UCD School of Psychology, University College Dublin, Dublin, Ireland; ^4^Konrad Lorenz University Foundation, Bogota, Colombia; ^5^Stress Prevention Unit, University of Sassari, Sassari, Italy; ^6^Department of Biomedical Sciences, University of Sassari, Sassari, Italy; ^7^STEM Unit and Centre for Workplace Excellence, University of South Australia, Adelaide, SA, Australia

**Keywords:** resilience, positivity, coping strategies, engagement-burnout, structural equation model, young adults

## Abstract

In a population of young adults, this study analyzes possible linear relations of resilience and positivity to coping strategies and engagement-burnout. The aim was to establish a model with linear, associative, and predictive relations, to identify needs and make proposals for therapeutic intervention in different student profiles. A population of 1,126 undergraduate students with different student profiles gave their informed, written consent, and completed validated questionnaires (CD-RISC Scale; Positivity; Coping Strategies of Stress; Engagement, and Burnout). An *ex post-facto* design involved bivariate association analyses, multiple regression and structural predictions. The results offered evidence of associations and predictive relationships between resilience factors, positivity, coping strategies and engagement-burnout. The factors of resilience and positivity had significant differential associations (positive and negative) with factors of coping strategies. Their negative relationship to burnout factors, and positive relation to engagement factors, is especially important. Results of structural analysis showed an acceptable model of relationships between variables. We conclude with practical implications for therapeutic intervention: (1) the proactive factors of resilience reflect a perception of self-efficacy and the ability to change adaptively; (2) the reactive factors of resilience are usually associated with withstanding experiences of change, uncertainty or trauma.

## Introduction

The problem of academic stress in the University context and the demands of therapeutic response in this context has had great relevance in recent times. Numerous recent investigations have analyzed mental health prevention strategies in young University students, in order to minimize the psychological effects of this situation (1, 2). To do this, they have focused their interest on the role of resilience and well-being. An example of this is the Monographic, in which this research is inserted (3).

The analysis of resilience, as a psychological variable in the sphere of preventive and therapeutic intervention, is important from both the structural and functional points of view (4–6). The distinction between structural and functional analysis of resilience is not often reflected in the previous literature, despite the importance of this distinction. *Structural analysis* of resilience makes it possible to reach a precise understanding of the role of each behavioral component of the theoretical construct, in order to infer therapeutic adjustment strategies for each person (7, 8). Questions that illustrate structural analysis could be: Do all components of resilience have the same functionality? Is it possible to identify certain components of resilience that have a proactive value and others that are more reactive in nature? In complementary fashion, *Functional analysis* contributes to a procedural view of the behaviors associated with each component of resilience, in relation to other variables (9). In this case, questions may refer to the most likely possible relationship between components of resilience and a given variable: What factors in resilience will be strongest in predicting the psychological variable positivity, or coping strategies? Positivity and coping strategies were selected as important behavioral factors that can help predict states of engagement vs. burnout, in the context of academic stress, just as previous research has suggested (10, 11). From an understanding of these structural and functional relationships, preventive and therapeutic intervention strategies can be plausibly established. The present study, therefore, offers a new model of evidence of plausible predictive relationships between the proactive and reactive components of resilience, positivity, coping strategies and state of engagement-burnout.

### Resilience and Mental Well-Being in Young Adults

Over the past 50 years, the psychological study of stress and resilience to adversity has been plentiful (12). With the influence of Positive Psychology, resilience has become a very popular topic in the field of psychopathology as well, where there is growing interest in positive adaptation in response to stress (13).

A recent meta-analysis by Grossman (14) has identified more than 10,000 articles that include the term resilience, relating it negatively to physical health complaints, and positively to overall well-being. Moreover, resilience has been positively associated with the experience of positive emotions and the use of adaptive coping strategies, that is, problem-focused coping (15). Most researchers agree on the general definition of resilience as the ability to withstand adversity or recover from stress and negative experiences (12, 14–17). Refining this definition, it can further be said that resilience is also the ability to move forward and grow in response to difficulties and challenges, that is, to become stronger through adversity (18).

The role of resilience, whether in protecting against stress, or in generating well-being, has been analyzed from several perspectives (19). Research also reports its value in personal recovery after health accidents (20), as well as in prevention of psychopathological symptoms, especially when resilience is worked on clinically within a cognitive-behavioral methodology (21). Additionally, recent studies have shown a connection between resilience and well-being, and between resilience and mental health (22), mediated by the relationship between optimism and subjective well-being (23, 24).

### Resilience and Behavioral Positivity as Protective Factors Against Stress

Resilience, as a personal characteristic, has been considered in Positive Psychology to be a factor that protects against stress (25). There is broad agreement that it is a complex, multidimensional construct (26). There is also consensus that two important aspects must be present to speak of resilience: an experience of adversity and a subsequent positive adaptation (13, 27–29). These two underlying aspects of resilient experience help us implicitly understand two types of resilient behavior: (1) *reactive*, bearing up under negative events, or the ability to withstand (30); recall as coined by Persius: “he conquers who endures”; and (2) *proactive*, or a reaction to events that actively seeks to restore well-being (31, 32); “look for the silver lining of the cloud” alludes to this type of behavior.

This positive adaptation brings benefits in terms of skills (hidden skills that are discovered and appreciated), relationships (which are selected, strengthened and improved), and changes in priorities and life philosophy, both toward the present and future (33). Moreover, scholars agree that resilience is an ability that can be the object of learning. Previous research points to the ability to bounce back as a relatively common phenomenon that does not stem from extraordinary qualities but from “ordinary magic” (34). Consequently, resilience improves with life experiences (35, 36). On the other hand, there is still much debate about its nature. There is no clear understanding or consensus in the scientific community about its structure or its components (14, 15), about the mechanisms that are implicit in the construct, or whether the processes and products of resilence should be considered traits or states (27, 37–41). Several recent studies have established the connection between resilience and mental health, through positivity (42). Yet to be established are the precise behavioral mechanisms by which resilience takes shape as behavior. The present study seeks to contribute toward this end.

### Resilience and Coping Strategies

Resilience has been associated with coping strategies, which have been identified as emotional meta-strategies (43, 44). Accordingly, resilience has been found to be associated with a positive predictor of self-regulation, learning approaches and coping strategies (45–47). A relationship has also been established with effective learning (48). The literature is clear in that resilience reflects successful management of stress events (49), moderating their negative effects, and promoting adaptation and psychological well-being (14, 29, 50).

Certain previous studies have established specific relationships between resilience and coping (39, 47). Resilience and coping are often used interchangeably, although there is growing evidence to suggest that they are conceptually distinct constructs, though related (37). Flecher and Srkar (27) indicate that “Resilience influences how an event is appraised whereas coping refers to the strategies employed following the appraisal of a stressful encounter” (p. 16). The message that emerges from the literature, according to these authors, is that resilience consists of various factors that promote personal assets and protect the individual from the negative appraisal of stressors; recovery and coping, then, are conceived as conceptually different from resilience.

Recent studies have shown that resilience and coping strategies are associated with and linearly predict well-being (51, 52), as well as different diseases and health problems (53, 54). Taking this consistent relationship further, the present study aims to show the mediational role of coping strategies between resilience and the motivational states of engagement-burnout.

### Resilience and the Emotional States of Engagement vs. Burnout

Resilience has appeared as a protective variable against stress, and a negative predictor (or protective) of burnout (55). In the sphere of employment, numerous studies have indicated a negative relationship between resilience and burnout (56), as well as a positive relationship with engagement (57). Other research studies have shown that emotional skills mediate in the states of engagement-burnout (58).

In the academic context, resilience has been considered as an attitudinal or meta-motivational variable, within the *Competence for Studing, learning and Performance with Stress*, a CSLS model of competence for managing academic stress [(59); in review]. Given its high degree of relationship with self-regulatory behavior, it has been conceptualized as a meta-ability that can determine the motivational state of students, in situations of academic stress. Therefore, it is possible to assume that it is a positive predictor of the motivational state of engagement and a negative predictor of the motivational state of burnout in University students. Several studies have reported the negative mediational role of resilience with respect to a state of burnout, and a positive mediational role in engagement (60, 61).

### Aims and Hypotheses

Yet to be established, however, are the specific mechanisms of how each component of resilience acts on the two motivational states (engagement vs. burnout), through coping strategies. This is the aim of the present study. Linear relations between resilience, coping strategies and engagement-burnout were applied to infer needs and proposals for intervening in different profiles of students. Based on prior evidence, the following hypotheses were posed: (H1) *resilience* would be associated with the personal variable of positivity, acting as a positive predictor; (H2) both variables, jointly, would be associated with and would be significantly positive predictors of *problem-focused strategies* and the motivational state of engagement; (H3) both would also be negative predictors of *emotion-focused strategies* and the motivational state of burnout.

## Methods

### Participants

An initial 1,126 undergraduate students participated in this study. The response rate was 95%, for a total of 1,069 students. This sample corresponds to a population of inference of 1,376 University students, with 99% total confidence and 0.1 percentage. The sample contained students enrolled in Psychology, Primary Education, and Educational Psychology; 85.5% were women and 14.5% were men. The age range was 19–25, and mean age was 21.33 years (sd = 2,73). Two Spanish public universities with similar characteristics were represented; 324 students attended one University and the remainder attended the other. The study design was incidental and non-randomized. The Guidance Department at each University invited teacher participation, and the teachers invited their own students to participate, on an anonymous, voluntary basis. Each course (subject) was considered one specific teaching-learning process.

### Instruments

#### Resilience

A validated Spanish version (62) of the *Connor-Davidson Resilience scale*, CD-RISC Scale (63) was used to measure resilience. Answers range from 1 (“Not true at all”) to 5 (“True nearly all the time”). Adequate reliability and validity values had been obtained in Spanish samples, and a five-factor structure emerged [Chi-square = 1,619, 170; Degrees of freedom (350-850) = 265; *p* < 0.001; Ch/Df = 6,110; SRMR (Standarized Root Mean-Square) = 0.062; NFI (Normed Fit Index) = 0.957; RFI (Relative Fix Index) = 0.948; IFI (Incremental Fix Index) = 0.922; TLI (Tucker Lewis index) = 0.980; CFI (Comparative fit index) = 0.920; RMSEA (Root Mean Square Error) = 0.063; HOELTER = 240 (*p* < 0.05) and 254 (*p* < 0.01)]. F1: Persistence/tenacity and strong sense of self-efficacy (TENACITY; alpha = 0.80); F2: Emotional and cognitive control under pressure (STRESS; alpha = 0.80); F3: Adaptability/ability to bounce back (CHANGE; alpha = 0.77); F4: Perceived Control (CONTROL; alpha = 0.77), and F5: Spirituality (alpha = 0.71).

#### Positivity

The positivity scale *Escala de Positividad*, by Caprara et al. (64), was used to measure this variable. Ten items are to be answered on a 5-point Likert scale. Acceptable values were obtained in our sample from the Spanish validation data [Chi-square = 208.992; Degrees of freedom (58-20) = 38; *p* < 0.001; Ch/Df = 5,499; SRMR (Standarized Root Mean-Square) = 0.062; NFI (Normed Fit Index) = 0.901; RFI (Relative Fix Index) = 0.894; IFI (Incremental Fix Index) = 0.912; TLI (Tucker Lewis index) = 0.923, CFI (Comparative fit index) = 0.916; RMSEA (Root Mean Square Error) = 0.085; HOELTER = 260 (*p* < 0.05) and 291 (*p* < 0.01)]. Good internal consistency was also found (Alpha = 0.893; Part 1 = 0.832, Part 2 = 0.813; Spearman-Brown = 0.862; Guttman = 0.832).

#### Coping Strategies

This variable was measured using the *Escala Estrategias de Coping* (Coping Strategies Scale), EEC, in its original version (65), validated for University students (66). Theoretical-rational criteria were used in constructing this scale, taking the Lazarus and Folkman questionnaire (67) and coping assessment studies by Moos and Billings (68) as foundational. Validation of the original, 90-item instrument produced a first-order structure with 64 items and a second-order structure with 10 factors and two dimensions, both of them significant. Answers range from 1 (“Not true at all”) to 5 (“True nearly all the time”). The second-order structure showed adequate fit values (Chi-square = 378.750; Degrees of freedom (87-34) = 53, *p* < 0.001; Ch/Df = 7,146; SRMR = 0.071; NFI = 0.901; RFI = 0.945; IFI = 0.903, TLI = 0.951, CFI = 0.903). Reliability was confirmed with the following measures: Cronbach alpha values of 0.93 (complete scale), 0.93 (first half) and 0.90 (second half), Spearman-Brown of 0.84 and Guttman 0.80. There are eleven factors and two dimensions: (1) Dimension: emotion-focused coping, F1. Fantasy distraction; F6. Help for action; F8. Preparing for the worst; F9. Venting and emotional isolation; F11. Resigned acceptance. (2) Dimension: problem-focused coping, F2. Help seeking and family counsel; F5. Self-instructions; F10. Positive reappraisal and firmness; F12. Communicating feelings and social support; F13. Seeking alternative reinforcement.

#### Engagement-Burnout

Adequate reliability and construct validity indices for this construct have been found in cross-cultural investigations. Engagement was assessed using a validated Spanish version of the *Utrecht Work Engagement Scale for Students* (69). Satisfactory psychometric properties were found with a sample of students from Spain. The model obtained good fit indices, and the second-order structure had three factors: vigor, dedication, and absorption. Answers range from 1 (“Not true at all”) to 5 (“True nearly all the time”). Scale unidimensionality and metric invariance were also confirmed in the samples assessed (Chi Square = 592.526, df = 74, *p* < 0.001; Ch/Df = 8,007; SRMR = 0.057; CFI = 0.954, TLI = 0.976, IFI = 0.954, TLI = 0.979, and CFI = 0.923; RMSEA = 0.083; HOELTER = 153, *p* < 0.05; 170 *p* < 0.01). The Cronbach alpha for this sample was 0.900 (14 items), with 0.856 (7 items) and 0.786 (7 items) for the two parts.

The Maslach Burnout Inventory, MBI (70), in its validated, open format Spanish version (69), was used to assess Burnout. Answers range from 1 (“Not true at all”) to 5 (“True nearly all the time”). Psychometric properties for this version were satisfactory in students from Spain. Good fit indices were obtained in this sample, and a second-order structure of three factors: exhaustion or depletion, cynicism, and lack of effectiveness. Scale unidimensionality and metric invariance were also confirmed in the samples assessed (Chi Square = 667.885, df = 87, *p* < 0.001; Ch/Df = 7,67; CFI = 0.956, TLI = 0.964, IFI = 0.951, TLI = 0.951, and CFI = 0.953; RMSEA = 0.071; HOELTER = 224, *p* < 0.05; 246 *p* < 0.01). The Cronbach alpha for this sample was 0.874 (15 items); the two parts of the scale showed 0.853 (8 items) and 0.793 (7 items), respectively.

### Procedure

In a single study, after signing their informed consent, students completed the validated questionnaires on an online platform. Scale completion was voluntary (71); students reported on five specific teaching-learning processes, each one representing a different University subject they took during a 2-year academic period. Presage variables were assessed in September-October of 2018 and 2019, Process variables in February-March of 2018 and 2019, and Product variables in May-June of 2018 and 2019. The respective Ethics Committees of the two universities approved the procedure, in the context of an R&D Project (2018-2021).

### Data Analyses

The *ex post-facto* design (72) of this cross-sectional study involved bivariate association analyses, multiple regresion and structural predictions (SEM). The preliminary analyzes were carried out to guarantee the adequacy in the use of the parametric analyzes carried out: normal distribution (Kolmogoroff-Sminorf), skewness and kurtosis (±0.05).

#### Correlation Analysis

In order to test the association hypotheses in H1, H2, and H3, we correlated positivity with the variable resilience, coping strategies, and engagement-burnout variables (Pearson bivariate correlation), using SPSS (v.25). The assumptions assumed and contrasted for the Pearson correlation were: (1) The data must have a linear relationship, this was determined through a scatter plot; (2) The variables must have a normal distribution; (3) The observations used for the analysis should be collected randomly from the reference population.

#### Prediction Analysis

For the prediction hypotheses of H1, H2, and H3, multiple regression analyses were carried out, and Beta indices of prediction and significance were calculated, using SPSS (v.25). The correlation and prediction factors were calculated using the factors originating from the exploratory factor analysis, prior to the confirmatory factor analysis.

#### Structural Equation Model

Two different Structural Equation Models (SEM) models were tested. In the first model, the effect of gender and the mediating prediction of engagement-burnout as predictors of coping strategies (Resilience → Positivity → Engagament-Burnout → Coping strategies) was evaluated; in the second model, the prediction presented in the graph and significantly valid (Resilience → Positivity → Coping strategies → Engagament-Burnout). Model fit was assessed by first examining the chi-square to degrees of freedom ratio as well as the Comparative Fit Index (CFI) and Normed Fit Index (NFI), Incremental Fit Index (IFI), and Relative Fit Index (RFI). These should ideally be >0.90. The Hoelter Index was also used to determine sample size adequacy (73). AMOS (v.26) was used for these analyses. Indirect effects values were assumed to be: the regression coefficients for small (0.14), medium (0.39), and large (0.59) effects are interpreted under the assumption that the error variances of the mediator and the dependent variable are both 1.0 (74). Direct, indirect and total effects, their significance levels and confidence intervals (75, 76) were calculated by bootstrapping (1,000 samples), using the maximum likelihood method (77). For the specific calculation of the confidence intervals of the indirect effects (Specific Indirect Effects mediation AMOS plugin, V.26) were used.

## Results

### Descriptive Preliminary Results

The direct and statistical values found in the preliminary sampling normality and adequacy tests showed acceptable values for the subsequent linear analysis of association and structural prediction carried out. See Table 1.

**Table 1:** Descriptive values of the analyzed variables.

**Variable**	**Minimum**	**Maximum**	**M**	**(Sd)**	**Statistical asymmetry**	**Asymmetry error desv**.	**Statistical Kurtosis**	**Kurtosis deviation**	**Kolmogorov-Smirnov statistical (*p*>)**
Resilience	1.82	4.86	3.74	(0.46)	−0.466	0.075	0.421	0.150	0.048 (0.200)
Positivity	1.25	5.00	3.76	(0.67)	−0.440	0.102	0.403	0.204	0.097 (0.976)
Emotional Coping	1.47	3.67	2.29	(0.31)	0.272	0.081	0.336	0.162	0.038 (0.994)
Problem Coping	1.09	3.29	2.50	(0.34)	−0.376	0.081	0.058	0.162	0.060 (0.979)
Burnout	1.00	4.78	2.22	(0.62)	0.483	0.069	0.318	0.137	0.072 (0.965)
Engagement	1.00	5.00	3.47	(0.66)	−0.215	0.069	0.302	0.139	0.053 (0.998)

### Bivariate Association Relations

#### Resilience and Positivity

The bivariate correlational analyses between resilience (total and factors) and positivity showed a significant positive association between the two, with particular associative strength for perceived control and tenacity. See Table 2.

**Table 2:** Bivariate correlations between resilience and positivity (*n* = 1,069).

**Criterion variable**	**Competence**	**Stress**	**Change**	**Control**	**Spirituality**	**Total**
Positivity	0.521^***^	0.300^***^	0.479^***^	0.576^***^	0.221^***^	0.592^***^

Competence: Self-efficacy/Tenacity; Stress: working under pressure; Change: adaptation to change and social support network; Control: perceived control; Spirituality: Beliefs and support in God. *p < 0.05; **p < 0.01;

*** *p < 0.001*.

#### Resilience and Coping Strategies

Bivariate correlational analyses between resilience (total and factors) and coping strategies showed several significant relationships. On one hand, the total resilience score was positively associated with total coping strategies (*r* = 0.245, *p* < 0.001). In general, all the factors or components of *resilience* appeared to be associated positively with coping strategies focused on the problem and negatively with factors focused on emotion, except for spirituality, which appeared positively associated with both. Specifically, this association was positive with problem-focused strategies (CF2. Seeking help and family advice; CF5. Self-Instructions; CF10. Positive reappraisal and firmness; CF12. Communicating feelings and social support; CF13. Seeking alternative reinforcement), and negative with emotion-focused strategies (CF8. Preparing for the worst; CF9. Emotional venting and isolation; CF11. Resigned acceptance). Three resilience factors followed this tendency, namely: perceived control (control), acceptance of change (change) and tenacity and perception of competence (competence). The tolerance to stress factor (stress) was low related to emotion-focused strategies (only with CF9. Emotional venting and isolation; CF11. Resigned acceptance). The only factor that was positively associated both with emotion-focused strategies and with problem-focused strategies was *spirituality* (CF1. Avoidant distraction; CF8. Preparing for the worst; CF11. Resigned acceptance). Of special interest is the negative association between the components of resilience and the CF9 factor (Emotional venting and isolation), as a precursor coping factor for health problems. See Table 3.

**Table 3:** Bivariate association of resilience with specific strategies for coping with stress (*n* = 1,069).

	**Competence**	**Stress**	**Change**	**Control**	**Spirituality**	**Total**
Emotion-focused coping	−0.163^***^	**−0.005**	−0.173^***^	−0.146^***^	0.145^***^	−0.069^*^
CF1	−0.011	−0.001	−0.024	0.014	0.197^***^	0.080^*^
CF7	−0.066^*^	−0.003	−0.056^*^	−0.105^***^	0.066^*^	−0.041
CF8	−0.101^**^	−0.018	−0.145^***^	−0.134^***^	0.103^***^	−0.068^*^
CF9	−0.301^***^	−0.099^*^	−0.300^***^	−0.322^***^	−0.031	−0.293^***^
CF11	−0.299^***^	−0.104^***^	−0.283^***^	−0.223^***^	0.074^*^	−0.208^***^
Problem-focused coping	0.316^***^	0.157^***^	0.315^***^	0.389^***^	0.229^***^	0.408^***^
CF2	0.133^***^	−0.054^*^	0.156^***^	0.301^***^	0.236^***^	0.257^***^
CF5	0.360^***^	0.330^***^	0.298^***^	0.235^***^	0.084^*^	0.231^***^
CF10	0.545^***^	0.480^***^	0.446^***^	0.345^***^	0.074^*^	0.491^***^
CF12	0.094^*^	−0.113^***^	0.149^***^	0.312^***^	0.187^***^	0.212^***^
CF13	0.179^***^	0.111^***^	0.143^***^	0.118^***^	0.149^***^	0.240^***^
Total	0.103^**^	0.087^**^	0.090^**^	0.171^**^	0.247^***^	0.245^***^

*Competence: Self-efficacy/Tenacity; Stress: working under pressure; Change: adaptation to change and social support network; Control: perceived control; Spirituality: Beliefs and support in God; Emotion-focused coping (D1): CF1. Avoidant distraction; CF7. Reducing anxiety and avoidance; CF8. Preparing for the worst; CF9. Emotional venting and isolation; CF11. Resigned acceptance; Problem-focused coping (D2): CF2. Seeking help and family advice; CF5. Self-Instructions; CF10. Positive reappraisal and firmness; CF12. Communicating feelings and social support; CF13. Seeking alternative reinforcement. Bold values: featured effects*.

* *p < 0.05*;

** *p < 0.01*;

*** *p < 0.001*.

#### Resilience and Engagement vs. Burnout

Total resilience was found to be consistently, significantly, and positively associated with *engagement* (*r* = 0.346*; p* < 0.001) and its components, and negatively with *burnout* (*r* = −0.372*; p* < 0.001) and its components, with particular associative strength for the component *lack of effectiveness*. Certain resilience factors were significantly associated with engagement and burnout, positively for the former, negatively for the latter: tenacity and perceived competence (*competence*), adaptation to change *(change)*, perceived control *(control)*, and stress tolerance (stress) were found to be positively associated with engagement; the component with the least associative strength was spiritual beliefs *(spirituality)*. Complementarily, the resilience factors that appeared negatively associated with burnout were tenacity and perceived competence *(competence)*, perceived control *(control)*, and adaptation to change (*change*). Moreover, the resilience factors that appeared negatively associated with burnout were the tenacity and perceived competence *(competence)*, perceived control *(control)*, and adaptation to change *(change);* with a lower associative force, the stress tolerance *(stress)* and spiritual beliefs *(spirituality)*. See Table 4.

**Table 4:** Bivariate associations of resilience and engagement-burnout (*n* = 1,069).

	**Competence**	**Stress**	**Change**	**Control**	**Spirituality**	**Resilience total**
Engagement	0.329^***^	0.233^***^	0.302^***^	0.294^***^	0.064^*^	0.346^***^
Vigor	0.344^***^	0.252^***^	0.304^***^	0.279^***^	0.047	0.345^***^
Dedication	0.258^***^	0.160^***^	0.243^***^	0.307^***^	0.067^*^	0.300^***^
Absorption	0.233^***^	0.176^***^	0.168^***^	0.168^***^	0.066^*^	0.234^***^
Burnout	−0.359^***^	−0.193^***^	−0.329^***^	−0.408^***^	−0.054^*^	−0.372^***^
Depletion	−0.280^***^	−0.155^***^	−0.258^***^	−0.317^***^	0.017	−0.266^***^
Cynicism	−0.196^***^	−0.65^*^	−0.197^***^	−0.320^***^	−0.076^*^	−0.247^***^
Lack of effectiveness	−0.454^***^	−0.293^***^	−0.395^***^	−0.379^***^	−0.065^*^	−0.430^***^

*Competence: Self-efficacy/Tenacity; Stress: working under pressure; Change: adaptation to change and social support network; Control: perceived control; Spirituality: Beliefs and support in God. Bold values: featured effects*.

* *p < 0.05*;

^**^p < 0.01;

*** *p < 0.001*.

### Multiple Prediction Relations

#### Resilience and Positivity

The multiple regression analysis showed a significant prediction effect of resilience factors on positivity. The resilience factors with the greatest positive predictive statistical effect were Perceived competence, Perceived control, and Spirituality. However, Tolerance to stress (stress) was not predictive of positivity. See Table 5.

**Table 5:** Regression relations between resilience components and positivity (*n* = 1,069).

**Criterion variable**	**Competence**	**Stress**	**Change**	**Control**	**Spirituality**	**Total**
Positivity	0.247^***^	−0.038	0.111^*^	0.367^***^	0.115^***^	*F*_(5, 974)_ = 50.149, *p* < 0.001, *R*^2^ = 0.405

*Competence: Self-efficacy/Tenacity; Stress: working under pressure; Change: adaptation to change and social support network; Control: perceived control; Spirituality: Beliefs and support in God*.

* *p < 0.05*;

^**^p < 0.01;

*** *p < 0.001*.

#### Resilience and Coping Strategies

Results of multiple regression showed three types of relations between resilience factors and coping strategies: (1) factors that negatively predicted the use of emotion-focused strategies and positively predicted problem-focused strategies: *perceived control, adaptation to change*, and *perceived competence*; (2) one factor that positively predicted the use of emotion-focused strategies and negatively predicted problem-focused strategies: *stress management*; (3) one factor that predicted the combined use of both strategy types: *Spirituality*.

It should be noted that in the case of emotion-focused strategies, the factors that were predicted with the most statistical force -significant and moderate correlation- were CF9 (*Emotional venting and isolation*) and CF11 *(Resigned acceptance*), while in problem-focused strategies, they were CF10 (Positive reappraisal and firmness), CF12 (Communicating feelings and social support), and CF5 (Self-Instructions). Of special note is Factor CF9, which was negatively predicted by the factors *perceived competence, perceived control* and *adaptation to change*. However, it was positively predicted by the *stress management* factor and unassociated with *spirituality*. See Table 6.

**Table 6:** Multiple regression of resilience to dimensions and factors of coping strategies (*n* = 1,069).

	**Competence**	**Stress**	**Change**	**Control**	**Spirituality**	**Total**
Coping total	0.025	0.047	−0.057	0.132^**^	0.216^***^	*F*_(5, 705)_ = 12.052^***^, *R*^2^ = 0.078
D1.Emotion-focused coping	−0.129^***^	0.171^***^	−0.172^***^	−0.078^*^	0.175^***^	*F*_(5, 839)_ = 16.028^***^, *R*^2^ = 0.087
CF1	−0.010	0.012	−0.057	0.004	0.207^***^	*F*_(5, 990)_ = 9.026^***^, *R*^2^ = 0.044
CF7	−0.035	0.058	−0.032	−0.102^**^	0.076^**^	*F*_(5, 990)_ = 3.770^***^, *R*^2^ = 0.019
CF8	−0.049	0.093^**^	−0.129^**^	0.096^**^	0.127^***^	*F*_(5, 990)_ = 9.805^***^, *R*^2^ = 0.048
CF9	−0.195^***^	0.149^***^	−0.150^***^	−0.193^***^	0.011	*F*_(5, 990)_ = 33.477^***^, *R*^2^ = 0.048
CF11	−0.246^***^	0.128^***^	−0.159^***^	−0.080^*^	0.118^*^	*F*_(5, 990)_ = 29.079^***^, *R*^2^ = 0.130
D2.Problem-focused coping	0.150^***^	−0.073^*^	0.104^**^	0.245^***^	0.161^**^	*F*_(5, 839)_ = 40.40^***^, *R*^2^ = 0.194
CF2	0.081^*^	−0.226^***^	0.061	0.265^***^	0.194^***^	*F*_(5, 992)_ = 35,321^***^, *R*^2^ = 0.156
CF5	0.197^***^	0.194^***^	0.033	0.045	0.023	*F*_(5, 992)_ = 36.102^***^, *R*^2^ = 0.157
CF10	0.331^***^	0.243^***^	0.089^**^	0.061^**^	−0.006	*F*_(5, 992)_ = 100.928^***^, *R*^2^ = 0.342
CF12	0.022	−0.281^***^	0.117^**^	0.290^***^	0.144^**^	*F*_(5, 992)_ = 39.556^***^, *R*^2^ = 0.166
CF13	0.109^**^	0.004	0.007	0.081	0.163^*^	*F*_(5, 992)_ = 13.356^***^, *R*^2^ = 0.065

*Competence: Self-efficacy/Tenacity; Stress: working under pressure; Change: adaptation to change and social support network; Control: perceived control; Spirituality: Beliefs and support in God; Emotion-focused coping (D1): CF1. Avoidant distraction; CF7. Reducing anxiety and avoidance; CF8. Preparing for the worst; CF9. Emotional venting and isolation; CF11. Resigned acceptance; Problem-focused coping (D2): CF2. Seeking help and family advice; CF5. Self-Instructions; CF10. Positive reappraisal and firmness; CF12. Communicating feelings and social support; CF13. Seeking alternative reinforcement*.

* *p < 0.05*;

** *p < 0.01*:

*** *p < 0.001*.

#### Resilience and Engagement-Burnout

Results of multiple regression showed three types of relations between resilience factors and the motivational state of engagement-burnout: (1) factors that negatively predicted burnout, and positively predicted engagement, as well as its components: *perceived competence, perceived control*, and *adaptation to change*. *Perceived competence* positively predicted, with greater strength, the components of vigor, dedication and absorption; *perceived control* was a significant negative predictor of the emotional state of depletion, cynicism and lack of effectiveness; *adaptation to change* had the same tendency, but with less strength; (2) two factors that did not significantly predict burnout and engagement: *tolerance of stress* and *spirituality*. The only factor that positively and significantly predicted depletion was *spirituality*. See Table 7.

**Table 7:** Multiple regression of resilience to engagement-burnout (*n* = 1,069).

	**Competence**	**Stress**	**Change**	**Control**	**Spirituality**	**Effect**
**Engagement**	0.200^***^	**0.038**	0.090^*^	0.152^***^	**0.007**	*F*_(5, 994)_ = 32.563^***^ *R*^2^ = 0.151
Vigor	0.223^***^	0.053	0.085^*^	0.132^***^	−0.011	*F*_(5, 994)_ = 36.637^***^ *R*^2^ = 0.158
Dedication	0.141^***^	0.010	0.048	0.206^***^	0.013	*F*_(5, 994)_ = 25.025^***^ *R*^2^ = 0.115
Absorption	0.139^***^	0.036	0.069	0.059	0.028	*F*_(5, 994)_ = 13.344^***^ *R*^2^ = 0.064
**Burnout**	−0.208^***^	**0.044**	−0.079^*^	−0.291^***^	**0.029**	*F*_(5, 994)_ = 49.636^***^ *R*^2^ = 0.208
Depletion	−0.169^***^	0.022	−0.036	−0.247^***^	0.082^**^	*F*_(5, 994)_ = 30.581^***^ *R*^2^ = 0.134
Cynism	−0.088^*^	0.084^*^	−0.038	−0.237^***^	−0.23	*F*_(5, 994)_ = 23.237^***^ *R*^2^ = 0.106
Lack of effectiveness	−0.282^***^	−0.024	−0.130^**^	−0.172^***^	0.016	*F*_(5, 994)_ = 64.540^***^ *R*^2^ = 0.249

*Competence: Self-efficacy/Tenacity; Stress: working under pressure; Change: adaptation to change and social support network; Control: perceived control; Spirituality: Beliefs and support in God. Bold values: featured effects*.

* *p < 0.05*;

** *p < 0.01*;

*** *p < 0.001*.

### Structural Prediction Model

**Figure 1:**
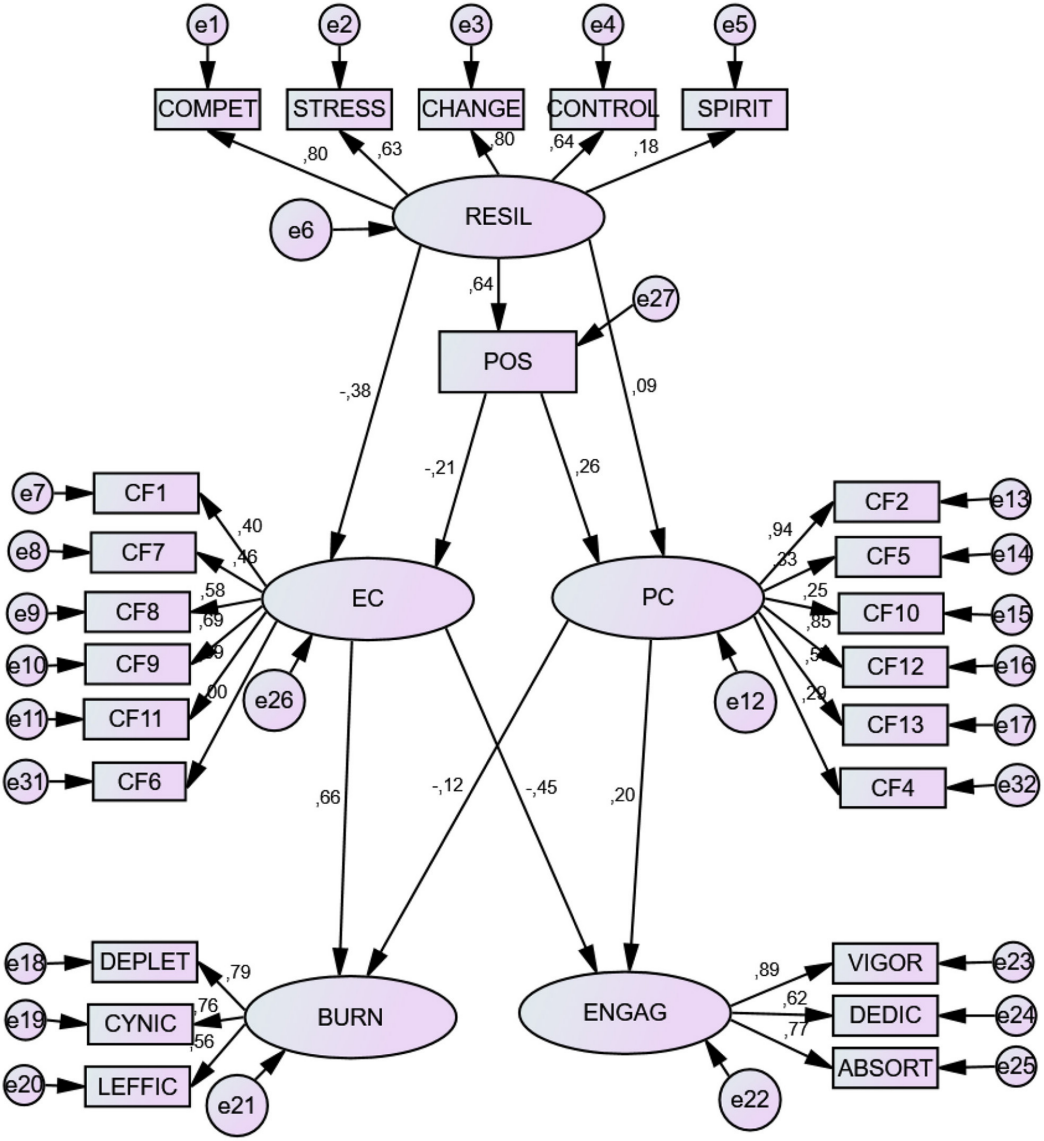
Structural prediction model. RESIL, resilience; POS, Positivity; EC, Emotional Coping; PC, Problem Coping; BURN, Burnout; ENGAG, Engagement. COMPET, Persistence/tenacity and strong sense of self-efficacy; STRESS, Emotional and cognitive control under pressure; CHANGE, Adaptability/ability to bounce back; CONTROL, Perceived Control; SPIRIT, Spirituality. Emotion-focused coping: F1. Avoidant distraction; F7. Reducing anxiety and avoidance; F8. Preparing for the worst; F9. Emotional venting and isolation; F11. Resigned acceptance; Problem-focused coping: F2. Seeking help and family advice; F5. Self-Instructions; F10. Positive reappraisal and firmness; F12. Communicating feelings and social support; F13. Seeking alternative reinforcement. DEPLET, depletion; CYNIC, Cynicism; LEFFIC, Lack of effectiveness; VIGOR, vigor; DEDIC, Dedication; ABSORT, Absorption.

Evidence was obtained of association and prediction relationships between resilience factors, coping strategies and engagement-burnout. Different significant associations (positive or negative) appeared between resilience factors and factors of coping strategies. The negative relationship to burnout factors, and positive relation to engagement factors, was especially important. The SEM results showed an acceptable relationship model. See Table 8 and Figure 1.

**Table 8:** Models of structural linear results of the variables (*n* = 1,069).

**Model**	**Chi square (*p* < 0.001)**	**DF**	**Chi/df CI**	**SMRM**	**NFI**	**RFI**	**TLI**	**CFI**	**RMSEA**	**Hoelter 05-01**
1	502.808	69	7.28	0.0728	0.917	0.907	0.920	0.900	0.080	175-194
2	1581.518	201	7.86	0.0686	0.926	0.937	0.935	0.928	0.081	206-213

*Model 1: Resilience → Engagement-Burnout → Coping Strategies; Model 2: Resilience → Coping Strategies → Engagement-Burnout*.

#### Direct Effects

There were several significant, direct prediction effects. Resilience showed a significant predictive effect on positivity. These two in conjunction appeared as positive predictors of *problem-focused coping* and negative predictors of *emotion-focused coping*. While *resilience* was the best negative predictor of *emotion-focused coping, positivity* was the best predictor of *problem-focused coping*. The factors that appeared with the most weight in the construct were *perceived competence, ability to adapt to change*, and *perceived control*.

*Problem-focused coping* was a positive predictor of engagement and negative predictor of burnout, while *emotion-focused coping* was a positive predictor *burnout* and negative predictor of *engagement*. F2 (Seeking help and family advice) and F12 (Communicating feelings and social support) were the factors with most weight in *problem-focused coping*, referring to social support; F11 (Resigned acceptance) and F9 (Emotional venting and isolation) had the most weight in *emotion-focused coping*.

*Absorption* and *vigor* were the factors with most weight in *engagement; depletion;* and *cynicism* had the most weight in *burnout* (See Table 9). Specific partial direct effects are shown in Table 10.

**Table 9:** Standardized direct effects (default model).

	**Resilience**	**Positivity**	**Problem-focused coping**	**Emotion-focused coping**	**Engagement**	**Burnout**
Positivity	0.664					
Problem-focused coping	0.090	0.256				
Emotion-focused coping	−0.379	−0.211				
Engagement			0.204	−0.446		
Burnout			−0.124	0.658		
Competence	0.802					
Stress	0.632					
Change	0.799					
Control	0.645					
Spirituality	0.176					
CF2			0.932			
CF5			0.331			
CF10			0.249			
CF12			0.851			
CF13			0.567			
CF1				0.405		
CF7				0.462		
CF8				0.557		
CF9				0.689		
CF11				0.694		
VIGOR					0.774	
DEDICAT					0.619	
ABSORP					0.872	
DEPLETI						0.795
CYNICISM						0.793
L. EFFEC						0.556

*Emotion-focused coping: F1. Avoidant distraction; F7. Reducing anxiety and avoidance; F8. Preparing for the worst; F9. Emotional venting and isolation; F11. Resigned acceptance; Problem-focused coping: F2. Seeking help and family advice; F5. Self-Instructions; F10. Positive reappraisal and firmness; F12. Communicating feelings and social support; F13. Seeking alternative reinforcement*.

**Table 10:** Direct effects specific and partial standardized values (95% B-CCI).

**Direct path**	**Unstandarized stimate**	**Lower**	**Upper**	***P*-Value**	**Standarized stimate**
RES → POS	0.643	0.224	0.723	0.001	0.664^***^
RES → PC	0.083	0.037	0.183	0.151	0.090
RES → EC	-0.361	0.312	0.581	0.001	−0.379^***^
POS → PC	0.223	0.147	0.348	0.01	0.256^**^
POS → EC	-0.162	−0.156	0.314	0.01	−0.211^**^
PC → ENG	0.217	0.182	0.316	0.01	0.204^**^
PC → BUR	-0.103	−0.083	0.215	0.01	−0.124^**^
EC → ENG	-0.389	−0.227	0.567	0.001	−0.446^***^
EC → BUR	0.579	0.221	0.743	0.001	0.658^***^

RES, Resilience; POS, Positivity; EC, Emotional Coping; PC, Problem Coping; ENG, Engagement; BUR, Burnout. *p < 0.051;

** *p < 0.01*;

*** *p < 0.001*.

#### Indirect Effects

There were several indirect positive effects of Resilience and Positivity. Both variables showed multiple predictive indirect effects, in the same direction as the direct effects. Likewise, Coping Strategies had indirect effects on the components of Engagement and of Burnout: *problem-focused strategies* showed positive effects on Engagement and negative effects on Burnout, while *emotion-focused strategies* had inverse effects. Specifically, *Resilience* indirectly and positively predicted F2 (Seeking help and family advice) and F12 (Communicating feelings and social support), and negatively F9 (Emotional venting and isolation) and F11 (Resigned acceptance). It also positively and indirectly predicted the components of engagement and negatively the components of burnout. In a complementary way, *Positivity* indirectly and positively predicted F2 (Seeking help and family advice) and F12 (Communicating feelings and social support), and negatively F8 (Preparing for the worst). Finally, the strategies focused on the *problem* had an indirect and positive predictive effect on the engagement factors and negative on the burnout factors; however, the strategies focused on *emotion* had the reverse, that is, an indirect positive prediction on burnout and negative on engagement (see Table 11). Specific partial indirect effects are shown in Table 12.

**Table 11:** Standardized indirect effects (default model).

	**Resilience**	**Positivity coping**	**Problem-focused coping**	**Emotion-focused**	**Engagement**	**Burnout**
Positivity						
Problem-focused coping	0.165					
Emotion-focused coping	−0.136					
Engagement	−0.282	0.146				
Burnout	−0.370	−0.171				
Competence						
Stress						
Change						
Control						
Spirituality						
CF2	0.239	0.240				
CF5	0.084	0.085				
CF10	0.063	0.064				
CF12	0.217	0.218				
CF13	0.145	0.143				
CF1	−0.208	−0.086				
CF7	−0.238	−0.098				
CF8	−0.297	−0.122				
CF9	−0.355	−0.146				
CF11	−0.357	−0.147				
VIGOR	0.251	0.113	0.182	−0.397		
DEDICAT	0.174	0.091	0.126	−0.276		
ABSORP	0.218	0.131	0.158	−0.345		
DEPLETI	−0.294	−0.136	−0.098	0.523		
CYNICISM	−0.281	−0.130	−0.094	0.499		
L. EFFEC	−0.206	−0.095	−0.069	0.366		

*Emotion-focused coping: F1. Avoidant distraction; F7. Reducing anxiety and avoidance; F8. Preparing for the worst; F9. Emotional venting and isolation; F11. Resigned acceptance; Problem-focused coping: F2. Seeking help and family advice; F5. Self-Instructions; F10. Positive reappraisal and firmness; F12.Communicating feelings and social support; F13. Seeking alternative reinforcement*.

**Table 12:** Indirect effects specific and partial standardized values (95% B-CCI).

**Indirect path**	**Unstandarized estimate**	**Lower**	**Upper**	***P*-Value**	**Standarized estimate**
RES → POS → EC	−0.142	−0.124	0.243	0.01	−0.136*
RES → POS → PC	0.175	0.048	0.274	0.01	0.165*
RES → EC → BUR	−0.363	−0.253	0.589	0.001	−0.370***
RES → PC → ENG	0.261	0.142	0.504	0.001	0.282***
POS → PC → ENG	0.140	0.047	0.057	0.01	0.146*
POS → PC → BUR	−0.162	−0.056	0.253	0.01	−0.171*

*RES, Resilience; POS, Positivity; EC, Emotional Coping; PC, Problem Coping; ENG, Engagement; BUR, Burnout*.

## Discussion

This study aimed to show the relationship between resilience, positivity, coping strategies and the emotional state of burnout or engagement in undergraduate students. This relationship has not been reported previously, and, furthermore, it allows us to infer various implications for therapeutic intervention in mental health. The results referring to bivariate linear associations (Hypothesis 1) gave empirical evidence that resilience and positivity scores maintain a significant, positive association (78–80), especially in the case of the components *perceived competence* (tenacity and self-efficacy) and *perceived control*. These results reinforce the idea that resilience involves an important perception of self-efficacy and self-control (25, 81–86). The results also agree with previous research that has shown a consistent relationship between self-regulation and resilience (10, 45, 87, 88). In other words, an outlook of positivity seems more likely when a person's learning history has equipped them with positive achievement experiences, based on a perception of ability when facing adversity (29, 89, 90).

In the case of the association between *resilience* and *coping strategies*, the results showed that resilience is associated with a greater number of coping strategies –positive association with problem-focused strategies and negative with emotion-focused– especially in the case of *perceived control, acceptance of change* and *perceived competence*. These results expand on and refine those found in prior evidence (84, 91–98), since the three behavioral factors would make the use of emotional management strategies less necessary; a higher level of self-regulation allows situations to be perceived with a lower level of stress (1, 10, 83–87, 89, 90, 93–100, 104, 105, 113–116, 122–125, 143–145, 148). It is noteworthy that the *stress tolerance* factor (stress) was less related to emotion-focused strategies, which also implies a lower level of perceived stress (101–103). Also worth mentioning is the *spirituality* factor, which was the only factor associated with both emotion-focused strategies and problem-focused strategies (104, 105). This would make it a kind of *catalyst* to other components which tend toward one type of strategy or another (106–108). Previous research has suggested the possibility that there are two different types of resilience profiles, with and without the spirituality factor (109–111).

The association between *resilience* and the motivational state of *engagement-burnout* appeared in the same direction as reported by previous research. In other words, there was a positive association with the state of *engagement* and a negative association with *burnout*, giving empirical value to resilience as a protective factor against stress (58, 112), by means of students' emotional state (11, 113–116).

In the case of multivariate prediction relationships (*Hypothesis 2*), the results allow us to refine previous association relationships. The resilience factors that best predicted *positivity* were perceived competence, perceived control, and spirituality, while tolerance of stress did not appear as a significant predictor of positivity. This relationship might suggest that resilience includes proactive factors (based on positivity) and reactive factors (stress tolerance). It is not the same to be proactively positive in the face of stress than to bear with it in a reactive way (117–121).

Predictive relationships in relation to coping strategies have reinforced a consistent view of their directionality (122–125). Once again, the factors of *perceived control, adaptation to change*, and *perceived competence* negatively predicted the use of emotion-focused strategies and positively predicted problem-focused strategies (47). The factor *tolerance to stress* positively predicted the use of emotion-focused strategies and negatively predicted problem-focused strategies. Special attention must be given to the use of strategy F9 (Emotional venting and isolation), due to its harmful effect on physical and psychological health (126). This might suggest that the resilience factor *tolerance to stress*, as a passive or reactive factor in stress management, may involve harmful components from the behavioral point of view (127). The *spirituality* factor, however, predicted the combined use of problem- and emotion-focused strategies, making it a factor that adds value to the previous resilience factors (110, 128).

Overall, the multivariate, linear predictive structural relationships (*Hypothesis 3*) confirmed the predictions proposed. Resilience was found to positively predict positivity, and these two together predict a double path of influence: (1) positively predicting the use of problem-focused strategies and engagement, (2) negatively predicting the use of emotion-focused strategies and burnout. These novel results identify the specific coping mechanisms in the direct and indirect influence of resilience on engagement and on burnout, complementing previous research (57, 129). However, identification of this relationship does not exhaust the possibilities of other influences and factors, which future research should establish.

It is also necessary to recognize certain *limitations* of the present investigation. First, there is the cross-sectional nature of the study. Second, the search for general models of relationships between these variables—already complex in itself- has meant setting aside the analysis of certain potentially mediating variables, such as gender and cultural diversity; previous research has established that both factors play a role (130, 131). Third, the use of self-report tools for collecting data is always a well-known risk of bias. Future research should combine different evaluation systems (132). Fourth, the sample is University standardized and not clinical; results should therefore be taken with caution, and any inferences toward the clinical population must be done in a contextualized way. Fifth, the sample is composed predominantly of women. Consequently, all these limitations should be resolved in future research studies, expanding the sample type and analyzing different profiles or clusters of resilience types (133). The connection to other important variables, such as socioeconomic status and personal strengths, should also be clarified and delimited, considering their importance in current research. It would therefore be of interest to establish relationships between character strengths and resilience (84, 134–136).

## Conclusions

The above results confirm prior evidence and add new detail regarding to the structure and functionality of the construct of resilience. The structural analyses allow us to state that there are different profiles of factors: (1) *proactive factors of resilience*, its core components, with greater positive, proactive value, such as perceived competence, perceived control, and adaptation to change. In all three cases, they reflect a perception of self-efficacy and the ability to adapt in changing environments (31, 137). (2) *reactive factors of resilience*, bearing with the negative emotion and maintaining the positive emotion that is usually associated with experiences of change, uncertainty or trauma (138); (3) the *catalyzing factor of resilience*, referring to *spirituality*, which adds value to the above factors, and may be considered a type of personal strength (139). This diversity of factors might indicate that there are different profiles of resilient persons, depending on the combination of the different factors in each person. Future research should inquire further into these complementary profiles.

### Implications

Regarding implications for the *practice of assessment* and *intervention in mental health, one* can reasonably infer that these variables ought to be assessed in processes of post-traumatic stress or traumatic experiences. These variables convey crucial information about relevant factors to understanding and that can be protective for young adults, making it possible to predict successful outcomes from such situations (107, 140). They also allow us to start from a previous explanatory model, and to infer factors for intervening at a molecular (clinical) level and at a molar (educational and contextualized) level of analysis (141).

Regarding *implications for the promotion of mental health* in higher education settings, in the Health, Counseling and Disability Services blog at Finders University, Garth Furber (142) indicates that Resilience is not an optional extra, not something that is nice to have, but something essential to build (143–145). The competency model for studying, learning, and performing under stress (SLPS competency) has considered resilience a meta-motivational variable, coping strategies to be meta-emotional variables, and engagement-burnout an emotional state that favors or hinders learning and academic achievement. The emotional fragility of students has become a serious problem in the university. Developing the capacity of resilience to stress is a precursor of student well-being (146, 147). Universities are recognizing its importance and are beginning to invest in research and services designed to build resilience. The specific relationships that have been demonstrated between these variables make it possible to design specific University intervention programs, all universities should have centers that offer counseling and psychological support for students (148). Also, the pandemic could represent an extra burden in this equation that is not accounted in this paper.

## Data Availability Statement

The raw data supporting the conclusions of this article will be made available by the authors, without undue reservation.

## Ethics Statement

The studies involving human participants were reviewed and approved by Comité de Ética de la Universidad de Navarra; http://www.estres.investigacion-psicopedagogica.org/lib/pdf/CERTIFICADO_COMITE_DE_ETICA_UNAV.pdf. The patients/participants provided their written informed consent to participate in this study.

## Author Contributions

JF: director of the project, conceptualization, data analysis, and first draft. FS and SP: critical review and writing. AG-U and SF: data collection, data analysis, and project support. GS: technical support for the project. All authors: contributed to the article and approved the submitted version.

## Conflict of Interest

The authors declare that the research was conducted in the absence of any commercial or financial relationships that could be construed as a potential conflict of interest.

## References

[1] de la Fuente J Paoloni PV Vera-Martínez MM Garzón-Umerenkova A. Effect of levels of self-regulation and situational stress on achievement emotions in undergraduate students: class, study and testing. Int J Environ Res Public Health. (2020) 17:4293. 10.3390/ijerph17124293 32560173 PMC7345223

[2] Nahum M Afek A Ben-Avraham R Davidov A Cohen NB Yehuda AB. *Psychological* resilience, mental health and inhibitory control among youth and young adults under stress. Front Psychol. (2021) 10:446. 10.3389/fpsyg.2019.00446 PMC7874000 33584372

[3] Gabrielli S Robis D Cefai C. Promoting resilience interventions for mental well-being in youth: research topic. Front Psychol. (2020). Available online at: https://www.frontiersin.org/research-topics/13113/promoting-resilience-interventions-for-mental-well-being-in-youth#articles 10.3389/fpsyt.2022.859546 PMC8888435 35250681

[4] Frangou S. Brain structural and functional correlates of resilience to bipolar disorder. Front Hum Neurosci. (2012) 5:184. 10.3389/fnhum.2011.00184 22363273 PMC3277296

[5] Matheson K Asokumar A Anisman H. Resilience: safety in the aftermath of traumatic stressor experiences. Front Behav Neurosci. (2020) 14:596919. 10.3389/fnbeh.2020.596919 33408619 PMC7779406

[6] Moreno-López L Ioannidis K Askelund AD Smith AJ Schueler K Van Harmelen AL. The resilient emotional brain: a scoping review of the medial prefrontal cortex and limbic structure and function in resilient adults with a history of childhood maltreatment. Biol Psychiatry Cogn Neurosci Neuroimaging. (2020) 5:392–402. 10.1016/j.bpsc.2019.12.008 32115373

[7] Askeland KG Hysing M Sivertsen B Breivik K. Factor structure and psychometric properties of the resilience scale for adolescents (READ). Assessment. (2020) 27:1575–87. 10.1177/1073191119832659 30818964

[8] Briganti G Linkowski P. Item and domain network structures of the resilience scale for adults in 675 University students. Epidemiol Psychiatr Sci. (2020) 29:1–9. 10.1017/S2045796020000323 31006419 PMC8061136

[9] Kasyanova E Vinogradova N. Resilience as a factor of professional development of railway engineering students. In: International Session of Factors of Regional Extensive Development (FRED-2019). Atlantis Press (2020). 10.2991/fred-19.2020.1

[10] de la Fuente J Amate J González-Torres MC Artuch R García-Torrecillas JM Fadda S. Effects of levels of self-regulation and regulatory teaching on strategies for coping with academic stress in undergraduate students. Front Psychol. (2020) 11:22. 10.3389/fpsyg.2020.00022 32082213 PMC7005059

[11] de la Fuente J Lahortiga-Ramos F Laspra-Solís C Maestro-Martín C Alustiza I Aubá E. Structural equation model of achievement emotions, coping strategies and engagement-burnout in undergraduate students: a possible underlying mechanism in facets of perfectionism. Int J Environ Res Public Health. (2020) 17:2106. 10.3390/ijerph17062106 PMC7143652 32235741

[12] Prince-Embury K Keefer KV Saklofske DH. Psychosocial skills: school-based promotion of resiliency in children and adolescents. In: Lipnevich AA Preckel F Roberts RD editors. Psychosocial Skills and School Systems in the 21st Century. Cham: Springer (2016). p. 301–24.

[13] O'Dougherty Wright M Masten AS Narayan AJ. Resilience processes in development: four waves of research on positive adaptation in the context of adversity. In: Goldstein S Brooks RB editors. Handbook of Resilience in Children. New York, NY: Springer (2013). p.15–37.

[14] Grossman MR. Clarifying the Nature of Resilience: A Meta-Analytic Approach (Graduate theses and dissertations) (2014). Available online at: http://scholarcommons.usf.edu/etd/5031 (accessed June 10, 2020).

[15] Grossman MR. The Structure of Resilience: An Empirical Examination of Resilience Factors (Graduate theses and dissertations). University of South Florida (2017). Retrieved from: https://scholarcommons.usf.edu/etd/6851 (accessed June 10, 2020).

[16] Gerson M Fernandez N. PATH: a program to build resilience and thriving in undergraduates. J Appl Soc Psychol. (2013) 43:2169–84. 10.1111/jasp.12168

[17] Petway KT Brenneman MW Kyllonen PC. Conecting noncognitive development to the Educational Pipeline. In: Khine MS Areepattamannil S editors. Non-cognitive Skills and Factors in Educational Attainment. Dordrecht: Sense Publishers (2016).

[18] Ryan J Jones S Hayes P Turner M. Building student resilience for graduate work readiness. In: Diver A editor. Employability via Higher education: Sustainabilility as Scholars. Liverpool: Springer (2019).

[19] Aburn G Gott M Hoare K. What is resilience? An integrative review of the empirical literature. J Adv Nurs. (2016) 72:980–1000. 10.1111/jan.12888 26748456

[20] Rapport LJ Wong CG Hanks RA. Resilience and well-being after traumatic brain injury. Disabil Rehabil. (2020) 42:2049–55. 10.1080/09638288.2018.1552327 31155974

[21] Chmitorz A Kunzler A Helmreich I Tüscher O Kalisch R Kubiak T. Intervention studies to foster resilience-a systematic review and proposal for a resilience framework in future intervention studies. Clin Psychol Rev. (2018) 59:78–100. 10.1016/j.cpr.2017.11.002 29167029

[22] Wu Y Sang Z Zhang X-C Margraf J. The relationship between resilience and mental health in Chinese college students: a longitudinal cross-lagged analysis. Front Psychol. (2020) 11:108. 10.3389/fpsyg.2020.00108 32116918 PMC7012791

[23] He F Cao R Feng Z Guan H Peng J. The impacts of dispositional optimism and psychological resilience on the subjective well-being of burn patients: a structural equation modelling analysis. PLoS ONE. (2013) 8:8–12. 10.1371/journal.pone.0082939 24358241 PMC3866201

[24] Miranda JO Cruz RNC. Resilience mediates the relationship between optimism and well-being among Filipino University students. Curr Psychol. (2020) 39:1–10. 10.1007/s12144-020-00806-0

[25] Hernandez AL González-Escobar S González NI López-Fuentes A Barcelata BE. Stress, self-efficacy, academic achievement and resilience in emerging adults. Electron J Res Educ Psychol. (2019) 17:129–48. 10.25115/ejrep.v17i47.2226

[26] Wu G Feder A Cohen H Kim JJ Calderon S Charney DS. Understanding resilience. Front Behav Neurosci. (2013) 7:10. 10.3389/fnbeh.2013.00010 23422934 PMC3573269

[27] Fletcher D Sarkar M. Psychological resilience: a review and critique of definitions, concepts, and theory. Eur Psychol. (2013) 18:12–23. 10.1027/1016-9040/a000124

[28] Masten AS. Resilience in developing systems: progress and promise as the fourth wave rises. Dev Psychopathol. (2007) 19:921–30. 10.1017/S0954579407000442 17705908

[29] Denovan A Crust L Clough PJ. Resilience at work. In: Oades LG Steger M Fave AD Passmore J editors. The Wiley Blackwell Handbook of the Psychology of Positivity and Strengths-Based Approaches at Work. Hoboken, NJ: John Wiley and Sons (2017). p. 132–49.

[30] Obradović J. How can the study of physiological reactivity contribute to our understanding of adversity and resilience processes in development? Dev Psychopathol. (2012) 24:371. 10.1017/S0954579412000053 22559120

[31] Holguin-Alvarez JA Rodríguez-Castillo MF. Proactividad y resiliencia en estudiantes emprendedores de Lima (Proactivity and resilience in entrepreneurial students from Lima). Propósitos y Representaciones. (2020) 8:1–20. 10.20511/pyr2020.v8n2.367

[32] Sierra MTC. Resiliencia, bienestar y aprendizaje a lo largo de la vida (Resilience, wellness and lifelong learning). Revista INFAD de Psicología. Int J Dev Educ Psychol. (2016) 1:161–70. 10.17060/ijodaep.2016.n2.v1.501

[33] Haidt J. The Happiness Hypothesis: Finding Modern Truth in Ancient Wisdom. New York, NY: Basic Books (2006).

[34] Masten AS. Ordinary Magic: Resilience in Development. New York, NY: Guilford Publications (2015).

[35] APA. The Road to Resilience. American Psychological Association (2014). Retrieved from: https://studentsuccess.unc.edu/files/2015/08/The-Road-to-Resiliency.pdf (accessed June 10, 2020).

[36] APA. The Road to Resilience. (2020). Retrieved from: https://www.apa.org/centrodeapoyo/resiliencia-camino (accessed June 10, 2020).

[37] Alonso-Tapia J Rodríguez-Rey R Garrido E Saiz H Ruiz M Nieto C. Coping, personality, and resilience: prediction of subjective resilience from coping strategies and protective personality factors. Behav Psychol Psicologí*a Conductual*. (2019) 27:375–89.

[38] Denovan A Dagnalla N Dhingrab K Grogana S. Evaluating the perceived stress scale among UK University students: implications for stress measurement and management. Stud Higher Educ. (2019) 44:120–33. 10.1080/03075079.2017.1340445

[39] Tamannaeifar M Shahmirzaei S. Prediction of academic resilience based on coping styles and personality traits. Pract Clin Psychol. (2019) 7:1–10. 10.32598/jpcp.7.1.1

[40] Johnson ML Taasoobshirazi G Kestler JL Cordova JR. Models and messengers of resilience: a theoretical model of college students' resilience, regulatory strategy use, and academic achievement. Educ Psychol. (2015) 35:869–85. 10.1080/01443410.2014.893560

[41] Liu H Zhang J Ji Y Yang L. Biological and psychological perspectives of resilience: is it possible to improve stress resistance? Front Hum Neurosci. (2018) 12:326. 10.3389/fnhum.2018.00326 30186127 PMC6110926

[42] Ungar M Theron L. Resilience and mental health: how multisystemic processes contribute to positive outcomes. Lancet Psychiatry. (2020) 7:441–8. 10.1016/S2215-0366(19)30434-1 31806473

[43] Ben-Zur H. The effectiveness of coping meta-strategies: perceived eficiency, emotional correlates and cognitive performance. Pers Individ Diff. (1999) 26:923–39. 10.1016/S0191-8869(98)00198-6

[44] de la Fuente J. Competency for Studying, Learning and Performing under Stress: Self-help guide for University students, graduates and professional examination candidates. Almería: Education and Psychology I+D+I: e-publishing RandDandI Series (2015).

[45] Artuch-Garde R González-Torres Md C de la Fuente J Vera MM Fernández-Cabezas M López-García M. Relationship between resilience and self-regulation: a study of Spanish youth at risk of social exclusion. Front Psychol. (2017) 8:612. 10.3389/fpsyg.2017.00612 28473792 PMC5397523

[46] de la Fuente J Zapata L Vera MM González-Torres MC Artuch-Garde R. Bullying, personal self-gulations, resilience, coping strategies and engagement-burnout: implications for intervention with universities students. In: Triggs P editor. Handbook of Bullying. New York, NY: Nova Science Publisher (2014). p. 91–107.

[47] de la Fuente J Fernández-Cabezas M Cambil M Vera MM González-Torres MC Artuch-Garde R. Linear relationship between resilience, learning approaches, and coping strategies to predict achievement in undergraduate students. Front Psychol. (2017) 8:1039. 10.3389/fpsyg.2017.01039 28713298 PMC5492468

[48] Prickett T Walters J Yang L Harvey M Crick T. Effective learning and resilience in first year undergraduate computer science. In: Proceedings of the 2020 ACM Conference on Innovation and Technology in Computer Science Education. New York, NY: Association for Computing Machinery (ACM) (2020). 10.1145/3341525.3387372

[49] Sarrionandia A Ramos-Díaz E Fernández-Lasarte O. Resilience as a mediator of emotional intelligence and perceived stress: a cross-country study. Front Psychol. (2018) 9:2653. 10.3389/fpsyg.2018.02653 30622503 PMC6308158

[50] Pidgeon AE Rowe NF Stapleton P Magyar HB Lo BC. Examining characteristics of resilience among University students: an international study. Open J Soc Sci. (2014) 2:14–22. 10.4236/jss.2014.211003

[51] Palma-Gómez A Herrero R Baños R García-Palacios A Castañeiras C Fernandez GL. Efficacy of a self-applied online program to promote resilience and coping skills in University students in four Spanish-speaking countries: study protocol for a randomized controlled trial. BMC Psychiatry. (2020) 148:1–15. 10.1186/s12888-020-02536-w PMC7133009 32248795

[52] Tomás JM Sancho P Melendez JC Mayordomo T. Resilience and coping as predictors of general well-being in the elderly: a structural equation modeling approach. Aging Mental Health. (2012) 16:317–26. 10.1080/13607863.2011.615737 22292552

[53] Lai HL Hung CM Chen CI Shih ML Huang CY. Resilience and coping styles as predictors of health outcomes in breast cancer patients: a structural equation modelling analysis. Eur J Cancer Care. (2020) 29:e13161. 10.1111/ecc.13161 31475417

[54] Tu PC Yeh DC Hsieh HC. Positive psychological changes after breast cancer diagnosis and treatment: the role of trait resilience and coping styles. J Psychosoc Oncol. (2020) 38:156–70. 10.1080/07347332.2019.1649337 31625826

[55] Fiorilli C Farina E Buonomo I Costa S Romano L Larcan R. Trait emotional intelligence and school burnout: the mediating role of resilience and academic anxiety in high school. Int J Environ Res Public Health. (2020) 17:3058. 10.3390/ijerph17093058 32354010 PMC7246930

[56] Anasori E Bayighomog SW Tanova C. Workplace bullying, psychological distress, resilience, mindfulness, and emotional exhaustion. Serv Ind J. (2020) 40:65–89. 10.1080/02642069.2019.1589456

[57] Malik P Garg P. Learning organization and work engagement: the mediating role of employee resilience. Int J Hum Resour Manag. (2020) 31:1071–94. 10.1080/09585192.2017.1396549

[58] Salmela-Aro K Upadyaya K. School engagement and school burnout profiles during high school-The role of socio-emotional skills. Eur J Dev Psychol. (2020) 17:1–22. 10.1080/17405629.2020.1785860

[59] de la Fuente J. A structural equation model of protection and risk factors for University academic stress: analysis and implications for the COVID-19 emergency. Front Psychol. (in review). 10.3389/fpsyg.2021.562372 PMC8415087 34484015

[60] Oyoo SA. Academic resilience as a predictor of academic burnout among form four students in Homa-Bay County, Kenya. Int J Educ Res. (2018) 6:187–200.

[61] Yu J Chae S. The mediating effect of resilience on the relationship between the academic burnout and psychological well-being of medical students. Korean J Med Educ. (2020) 32:13. 10.3946/kjme.2020.149 32130847 PMC7066430

[62] Manzano-García G Ayala-Calvo JC. New perspectives: towards an integration of the concept “burnout” and its explanatory models. Anal Psicol. (2013) 29:800–9. 10.6018/analesps.29.3.145241

[63] Connor KM Davidson JR. Development of a new resilience scale: the Connor-Davidson resilience scale (CD-RISC). Depress Anxiety. (2003) 18:76–82. 10.1002/da.10113 12964174

[64] Caprara GV Alessandri G Eisenberg N Kupfer A Steca P Caprara MG. The positivity scale. Psychol Assess. (2012) 24:701–12. 10.1037/a0026681 22250591

[65] Chorot P Sandín B. Escalas de Estrategias de Coping [Scales of Coping Strategies]. Madrid: UNED (1987).

[66] de la Fuente J. Competence for Studying, Learning and Performance Under Stress: Self-Help Guide for University Students, Graduates and Professional Examination Candidades. Almería: Education and Psychology I+D+I (2015).

[67] Lazarus RS Folkman S. Stress, Appraisal, and Coping. New York, NY: Springer (1984).

[68] Billings AC Moos RH. Psychosocial theory and research on depression: an integrative framework and review. Clin Psychol Rev. (1982) 2:213–37. 10.1016/0272-7358(82)90013-7

[69] Schaufeli WB Salanova M González-Romá V Bakker AB. The measurement of engagement and burnout: a two sample confirmatory factor analytic approach. J Happiness Stud. (2002) 3:71–92. 10.1023/A:1015630930326

[70] Maslach C Jackson SE Leiter MP. MBI: Maslach Burnout Inventory. Sunnyvale, CA: CPP (1996).

[71] de la Fuente J López M Zapata L Sollinas G Fadda S. Improving mental health trough and online self-assessment and self-help e-Utility in university students. In: Nata RV editor. Progress in Education, Vol. 33. New York, NY: Nova Publisher (2015). p. 63–74.

[72] Ato M Ato López J Benavente A. Un sistema de clasificación de los diseños de investigación en psicología (A classification system for research designs in psychology). Anales de Psicologí*a*. (2013) 29:1038–59. 10.6018/analesps.29.3.178511

[73] Tabachnick BG Fidell LS. SAS for Windows Workbook for Tabachnick and Fidell Using Multivariate Statistics. Allyn and Bacon1 (2001).

[74] Cheung MW-L. Comparison of methods for constructing confidence intervals of standardized indirect effects. Behav Res Methods. (2009) 41:425–38. 10.3758/BRM.41.2.425 19363183

[75] MacKinnon D Fairchild A Fritz M. Mediation analysis. Ann Rev Psychol. (2007) 58:593–614. 10.1146/annurev.psych.58.110405.085542 16968208 PMC2819368

[76] Preacher KJ Zhang Z Zyphur MJ. Alternative methods for assessing mediation in multilevel data: the advantages of multilevel SEM. Struct Equ Model. (2011) 18:161–82. 10.1080/10705511.2011.557329

[77] Rucker D Preacher K Tormala Z Petty R. Mediation analysis in social psychology: current practices and new recommendations. Soc Pers Psychol Compass. (2011) 5:359–71. 10.1111/j.1751-9004.2011.00355.x

[78] Bingöl TY Batik MV Hosoglu R Firinci Kodaz A. Psychological resilience and positivity as predictors of self-efficacy. Asian J Educ Train. (2019) 5:63–9. 10.20448/journal.522.2019.51.63.69

[79] Chambers C Ryder E. Supporting Compassionate Healthcare Practice: Understanding the Role of Resilience, Positivity and Wellbeing. Abingdon: Routledge. (2018).

[80] Milioni M Alessandri G Eisenberg N Caprara GV. The role of positivity as predictor of ego-resiliency from adolescence to young adulthood. Pers Ind Diff. (2016) 101:306–11. 10.1016/j.paid.2016.06.025

[81] Gomez-Baya D Tomé G Reis M Gaspar de Matos M. Long-term self-regulation moderates the role of internal resources for resilience in positive youth development in Portugal. J Genet Psychol. (2020) 181:127–49. 10.1080/00221325.2020.1735986 32151204

[82] Lin M Wolke D Schneider S Margraf J. Bullying history and mental health in University students: the mediator roles of social support, personal resilience, and self-efficacy. Front Psychiatry. (2020) 10:960. 10.3389/fpsyt.2019.00960 31993000 PMC6971115

[83] Freire C del Mar Ferradás M Regueiro B Rodríguez S Valle A Núñez JC. Coping strategies and self-efficacy in University students: a person-centered approach. Front Psychol. (2020) 11:841. 10.3389/fpsyg.2020.00841 32508707 PMC7248269

[84] Smith KJ Haight TD Emerson DJ Mauldin S Wood BG. Resilience as a coping strategy for reducing departure intentions of accounting students. Account Educ. (2020) 29:77–108. 10.1080/09639284.2019.1700140

[85] Bonanno GA Burton CL. Regulatory flexibility: an individual differences perspective on coping and emotion regulation. Perspect Psychol Sci. (2013) 8:591–612. 10.1177/1745691613504116 26173226

[86] Freire C Ferradás MM Núñez JC Valle A Vallejo G. Eudaimonic well-being and coping with stress in University students: the mediating/moderating role of self-efficacy. Int J Environ Res Public Health. (2019) 16:48. 10.3390/ijerph16010048 30585237 PMC6339215

[87] de la Fuente J Mañas I Franco C Cangas AJ Soriano E. Differential effect of level of self-regulation and mindfulness training on coping strategies used by University students. Int J Environ Res Public Health. (2018) 15:E2230. 10.3390/ijerph15102230 30314383 PMC6210926

[88] Freire C Ferradás MM Núñez JC Valle A. Coping flexibility and eudaimonic well-being in University students. Scand J Psychol. (2018) 59:433–42. 10.1111/sjop.12458 29852527

[89] DeRosier ME Frank E Schwartz V Leary KA. The potential role of resilience education for preventing mental health problems for college students. Psychiatr Ann. (2013) 43:538–44. 10.3928/00485713-20131206-05

[90] KarimiJozestani L Faramarzi S Yarmohammadian A. The effectiveness of training metacognition-based study skill on the students' achievement motivation, self-efficacy, satisfaction with school and resilience. Interdiscip J Virtual Learn Med Sci. (2020) 7:98–109. 10.5812/ijvlms.12151

[91] Secades XG Molinero O Salguero A Barquín RR de la Vega R Márquez S. Relationship between resilience and coping strategies in competitive sport. Percept Motor Skills. (2016) 122:336–49. 10.1177/0031512516631056 27420325

[92] Shing EZ Jayawickreme E Waugh CE. Contextual positive coping as a factor contributing to resilience after disasters. J Clin Psychol. (2016) 72:1287–306. 10.1002/jclp.22327 27410521

[93] Zimmer-Gembeck MJ Skinner EA. The development of coping: implications for psychopathology and resilience. In: Cicchetti D editor. Developmental Psychology: Risk, Resilience, and Intervention. New York, NY: Hoboken, NJ: John Wiley and Sons (2016). p. 485–545.

[94] Bettis AH Coiro MJ England J Murphy LK Zelkowitz RL Dejardins L. Comparison of two approaches to prevention of mental health problems in college students: enhancing coping and executive function skills. J Am Coll Health. (2017) 65:313–22. 10.1080/07448481.2017.1312411 28358274

[95] Chou P-C Chao Y-MY Yang H-J Yeh G-L Lee TS-H. Relationships between stress, coping and depressive symptoms among overseas University preparatory Chinese students: a cross-sectional study. BMC AQQ22Public Health. (2011) 11:352. 10.1186/1471-2458-11-352 21595974 PMC3118242

[96] Houston JB First J Spialek ML Sorenson ME Mills-Sandoval T Lockett M. Randomized controlled trial of the resilience and coping intervention (RCI) with undergraduate University students. J Am Coll Health. (2017) 65:1–9. 10.1080/07448481.2016.1227826 27559857

[97] Howard DE Schiraldi G Pineda A Campanella R. Stress and mental health among college students: overview and promising prevention interventions. In: Landow MV editor. Stress and Mental Health of College Students. New York, NY: Nova Science Publishers (2006). p. 91–124.

[98] Prince-Embury S Saklofske DH Keefer KV. Three-factor model of personal resiliency. In: Kumar U editor. Routledge International Handbooks. The Routledge International Handbook of Psychosocial Resilience. Abingdon: Routledge/Taylor and Francis Group (2017). p. 34–45.

[99] Cheng C Kogan A Chio JH. The effectiveness of a new, coping flexibility intervention as compared with a cognitive-behavioural intervention in managing work stress. Work Stress. (2012) 26:272–88. 10.1080/02678373.2012.710369

[100] Kobylińska D Kusev P. Flexible emotion regulation: how situational demands and individual differences influence the effectiveness of regulatory strategies. Front Psychol. (2019) 10:72. 10.3389/fpsyg.2019.00072 30774610 PMC6367256

[101] Friborg O Hjemdal O Rosenvinge JH Martinussen M Aslaksen PM Flaten MA. Resilience as a moderator of pain and stress. J Psychosom Res. (2006) 61:213–9. 10.1016/j.jpsychores.2005.12.007 16880024

[102] Lee CM Watson REB Kleyn CE. The impact of perceived stress on skin ageing. J Eur Acad Dermatol Venereol. (2020) 34:54–8. 10.1111/jdv.15865 31407395

[103] Shi X Wu J. Chronic stress and anticipatory event-related potentials: the moderating role of resilience. Stress. (2020) 23:607–13. 10.1080/10253890.2020.1766019 32401112

[104] Mpofu S Mabvurira V Chirimambowa T. Religion, spirituality and resilience of HIV positive children in Zimbabwe. Can Soc Sci. (2020) 16:1–10.

[105] Martínez-Rodríguez RDC Benítez-Corona L. Resilient coping strategies for physical therapy classes in Pachuca. In: Mazurek H editor. Pratiques Basées sur la Résilience. Hidalgo: AMU, IRD, LED. (2020). pp. 485–492.

[106] Borji M Memaryan N Khorrami Z Farshadnia E Sadighpour M. Spiritual health and resilience among University students: the mediating role of self-esteem. Pastoral Psychol. (2020) 69:1–10. 10.1007/s11089-019-00889-y

[107] Sadeghifard YZ Veisani Y Mohamadian F Azizifar A Naghipour S Aibod S. Relationship between aggression and individual resilience with the mediating role of spirituality in academic students-a path analysis. J Educ Health Promot. (2020) 9:2. 10.4103/jehp.jehp_324_19 32154297 PMC7032025

[108] Schulenberg SE editor. Positive Psychological Approaches to Disaster: Meaning, Resilience, and Posttraumatic Growth. London: Springer Nature (2020).

[109] González-Torres MC Artuch R. Perfiles de resiliencia y estrategias de afrontamiento en la universidad: variables contextuales y demográficas [Resilience profiles and coping strategies at university: contextual and demographic variables]. Electron J Res Educ Psychol. (2014) 12:621–48. 10.14204/ejrep.34.14032

[110] Mujib A Rena S. The Moderating Effect of Spirituality on the Relationship Between Academic Life Stressors and Perceived Stress in Medical Undergraduate Students. Jakarta: ICRMH (2019).

[111] Shrivastava A. Spiritual and non spiritual practices for work stress coping: a comparative study among academic faculties in india. Int J Indian Psychol. (2020) 8:1055–60. 10.25215/0801.133

[112] Smith NA Brown JL Tran T Suárez-Orozco C. Parents, friends and immigrant youths' academic engagement: a mediation analysis. Int J Psychol. (2020) 55:743–53. 10.1002/ijop.12672 32285451

[113] Turner J Bartlett D Andiappan M Cabot L. Students' perceived stress and perception of barriers to effective study: impact on academic performance in examinations. Br Dent J. (2015) 219:453–8. 10.1038/sj.bdj.2015.850 26564362

[114] Gustems-Carnicer J Calderón C Calderón-Garrido D. Stress, coping strategies and academic achievement in teacher education students. Eur J Teach Educ. (2019) 42:375–90. 10.1080/02619768.2019.1576629

[115] Vizoso CM Arias O. Estresores académicos percibidos por estudiantes universitarios y su relación con el burnout y el rendimiento académicos (Academic stressors perceived by University students and their relationship with academic burnout, efficacy and performance). Anu Psicol. (2016) 46:90–7. 10.1016/j.anpsic.2016.07.006

[116] González-Cabanach R Souto-Gestal A González-Doniz L Franco V. Perfiles de afrontamiento y estrés académico en estudiantes universitarios (Profiles of coping and academic stress among University students). Rev Invest Educ. (2018) 36:421–433. 10.6018/rie.36.2.290901

[117] Arampatzi E Burger M Stavropoulos S Tay L. The role of positive expectations for resilience to adverse events: subjective well-being before, during and after the Greek bailout referendum. J Happiness Stud. (2020) 21:965–95. 10.1007/s10902-019-00115-9

[118] Cruickshank N. He who defends everything, defends nothing: proactivity in organizational resilience. Transnational Corporations Rev. (2020) 12:1–11. 10.1080/19186444.2020.1764326

[119] Galiana DR. Análisis de la felicidad, resiliencia y optimismo como factores emocionales en la inserción laboral de los universitarios (tesis doctoral). España: Universidad Miguel Hernández De Elche (2015)

[120] Hadi S. New perspective on the resilience of SMEs proactive, adaptive, reactive from business turbulence: a systematic review. J Xi'an Univ Arch Technol. (2020) 12:1265–75.

[121] Jia X Chowdhury M Prayag G Chowdhury MMH. The role of social capital on proactive and reactive resilience of organizations post-disaster. Int J Disaster Risk Reduct. (2020) 48:101614. 10.1016/j.ijdrr.2020.101614

[122] Beiter R Nash R McCrady M Rhoades D Linscomb M Clarahan M. The prevalence and correlates of depression, anxiety, and stress in a sample of college students. J Affect Disord. (2015) 173:90–6. 10.1016/j.jad.2014.10.054 25462401

[123] Ethridge P Ali N Racine SE Pruessner J Weinberg A. Risk and resilience in an acute stress paradigm: evidence from salivary cortisol and time-frequency analysis of the reward positivity. Clin Psychol Sci. (2020) 8:872–89. 10.1177/2167702620917463

[124] Cabanach RG Valle A Rodríguez S Piñeiro I Freire C. Escala de Afrontamiento del Estrés Académico (A-CEA) (The coping scale of academic stress questionnaire (A-CEA)). Rev Iberoam Psicol Salud. (2010) 1:51–64.

[125] Tavolacci MP Ladner J Grigioni S Richard L Villet H Dechelotte P. Prevalence and association of perceived stress, substance use and behavioral addictions: a cross-sectional study among University students in France, 2009-2011. BMC Public Health. (2013) 13:724. 10.1186/1471-2458-13-724 23919651 PMC3750571

[126] Shoua-Desmarais N von Harscher H Rivera M Felix T Havas N Rodriguez P. First year burnout and coping in one US medical school. Acad Psychiatry. (2020) 44:394–8. 10.1007/s40596-020-01198-w 32130687

[127] Jiang H Jiang X Sun P Li X. Coping with workplace ostracism: the roles of emotional exhaustion and resilience in deviant behavior. Manag Decis. (2020) 59:358–71. 10.1108/MD-06-2019-0848

[128] Wiese-Bjornstal DM Wood KN Wambach AJ White AC Rubio VJ. Exploring religiosity and spirituality in coping with sport injuries. J Clin Sport Psychol. (2020) 14:68–87. 10.1123/jcsp.2018-0009

[129] Holliday KN. An Examination of the Impact of Mentoring on Girls Academic Engagement and Resilience (doctoral thesis), Texas State University, San Marcos, TX, United States (2020). Available online at: https://digital.library.txstate.edu/handle/10877/9871

[130] Verrochi D. Building resilience in gender and sexual minority youth. Creat Nurs. (2020) 26:109–13. 10.1891/CRNR-D-19-00047 32321794

[131] Alessi EJ Greenfield B Manning D Dank M. Victimization and resilience among sexual and gender minority homeless youth engaging in survival sex. J Interpers Violence. (2020) 36:1–24. 10.1177/0886260519898434 31920163

[132] Didkowsky N Ungar M Liebenberg L. Using visual methods to capture embedded processes of resilience for youth across cultures and contexts. J Can Acad Child Adolesc Psychiatry. (2010) 19:12–8. 20119562 PMC2809441

[133] Yu N. Using systemizing-empathizing theory to explore individual differences in resilience by brain types. In: International Conference on Mental Health and Humanities Education (ICMHHE 2020). Wuhan: Atlantis Press (2020). p. 68–78. 10.2991/assehr.k.200425.015

[134] Botha T. Flourishing Beyond Borders: Character Strengths, Resilience and Self-Perceived Well-Being of the Accompanying Expatriate Partner During International Relocation (doctoral dissertation). Potchefstroom: North-West University (2020).

[135] Florin M Schrimmer L McCargo S Bohn T Caton C. Fostering Hope and Enhancing Resilience through Character Strengths Interventions. (2020). Available online at: https://repository.upenn.edu/mapp_slp/35 (accessed July 15, 2020).

[136] Karris-Bachik MA Carey G Craighead WE. VIA character strengths among US college students and their associations with happiness, well-being, resiliency, academic success and psychopathology. J Posit Psychol. (2020) 15:1–14. 10.1080/17439760.2020.1752785

[137] Dehnad V. A proactive model to control reactive behaviors. World J Educ. (2017) 7:24–31. 10.5430/wje.v7n4p24

[138] Chen C. The role of resilience and coping styles in subjective well-being among Chinese University students. Asia Pacific Educ Res. (2016) 25:377–87. 10.1007/s40299-016-0274-5

[139] Porobić S. Long-term adaptation among naturalised bosnian refugees in sweden-existential preoccupation, spirituality and resilience. In: Forced Migration and Resilience. Wiesbaden: Springer (2020). p. 71–97.

[140] Gibbs LAL Anderson MI Simpson GK Jones KF. Spirituality and resilience among family caregivers of survivors of stroke: a scoping review. NeuroRehabilitation. (2020) 46:41–52. 10.3233/NRE-192946 32039873

[141] de la Fuente J González-Torres MC Aznárez-Sanado M Martínez-Vicente JM Peralta-Sánchez FJ Vera MM. Implications of unconnected micro, molecular, and molar level research in psychology: the case of executive functions, self-regulation, and external regulation. Front Psychol. (2019) 10:1919. 10.3389/fpsyg.2019.01919 31507487 PMC6719524

[142] Furber G. Disability Services blog at Finders University, (2018). Available online at: https://blogs.flinders.edu.au/student-health-and-well-being/2018/01/22/disability-services-seeking-mentors-new-students-disabilities/ (accessed December 6, 2020).

[143] Scharp KM Dorrance Hall E. Examining the relationship between undergraduate student parent social support-seeking factors, stress, and somatic symptoms: a two-model comparison of direct and indirect effects. Health Commun. (2019) 34:54–64. 10.1080/10410236.2017.1384427 29083239

[144] Cheng C Lau H-PB Chan MPS. Coping flexibility and psychological adjustment to stressful life changes: a meta-analytic review. Psychol Bull. (2014) 140:1582–607. 10.1037/a0037913 25222637

[145] Deasy C Coughlan B Pironom J Jourdan D Mannix-McNamara P. Psychological distress and coping amongst higher education students: a mixed method enquiry. PLoS ONE. (2014) 9:e115193. 10.1371/journal.pone.0115193 25506825 PMC4266678

[146] Beerten-Duijkers JC Vissers CTW Rinck M Barkley RA Egger OI. Self-directedness positively contributes to resilience and quality of life: findings from a mixed psychiatric sample. J Soc Clin Psychol. (2020) 39:59–76. 10.1521/jscp.2020.39.01.002

[147] Turner M Holdsworth S Scott-Young C. Resilience at University: the development and testing of a new measure. Higher Educ Res Dev. (2017) 36:386–400 10.1080/07294360.2016.1185398

[148] de la Fuente J Martínez-Vicente JM Peralta-Sánchez FJ González-Torres MC Artuch R Garzón-Umerenkova A. Satisfaction with the self-assessment of University students through e-Coping with academic stress Utility TM. Front Psychol. (2018) 9:1932. 10.3389/fpsyg.2018.01932 30467485 PMC6236068

